# The preparation temperature influences the physicochemical nature and activity of nanoceria

**DOI:** 10.3762/bjnano.12.43

**Published:** 2021-06-04

**Authors:** Robert A Yokel, Wendel Wohlleben, Johannes Georg Keller, Matthew L Hancock, Jason M Unrine, D Allan Butterfield, Eric A Grulke

**Affiliations:** 1 Pharmaceutical Sciences, University of Kentucky, Lexington, Kentucky, 40536-0596, USA; 2 BASF, 67056 Ludwigshafen am Rhein, Germany; 3 Chemical and Materials Engineering, University of Kentucky, Lexington, Kentucky, 40506-0046, USA; 4 Plant and Soil Sciences, University of Kentucky, Lexington, Kentucky, 40546-0091, USA; 5 Chemistry, University of Kentucky, Lexington, Kentucky, 40506-0055, USA

**Keywords:** cerium, dissolution, nanoparticles, physicochemical properties, valence state

## Abstract

Cerium oxide nanoparticles, so-called nanoceria, are engineered nanomaterials prepared by many methods that result in products with varying physicochemical properties and applications. Those used industrially are often calcined, an example is NM-212. Other nanoceria have beneficial pharmaceutical properties and are often prepared by solvothermal synthesis. Solvothermally synthesized nanoceria dissolve in acidic environments, accelerated by carboxylic acids. NM-212 dissolution has been reported to be minimal. To gain insight into the role of high-temperature exposure on nanoceria dissolution, product susceptibility to carboxylic acid-accelerated dissolution, and its effect on biological and catalytic properties of nanoceria, the dissolution of NM-212, a solvothermally synthesized nanoceria material, and a calcined form of the solvothermally synthesized nanoceria material (ca. 40, 4, and 40 nm diameter, respectively) was investigated. Two dissolution methods were employed. Dissolution of NM-212 and the calcined nanoceria was much slower than that of the non-calcined form. The decreased solubility was attributed to an increased amount of surface Ce^4+^ species induced by the high temperature. Carboxylic acids doubled the very low dissolution rate of NM-212. Nanoceria dissolution releases Ce^3+^ ions, which, with phosphate, form insoluble cerium phosphate in vivo. The addition of immobilized phosphates did not accelerate nanoceria dissolution, suggesting that the Ce^3+^ ion release during nanoceria dissolution was phosphate-independent. Smaller particles resulting from partial nanoceria dissolution led to less cellular protein carbonyl formation, attributed to an increased amount of surface Ce^3+^ species. Surface reactivity was greater for the solvothermally synthesized nanoceria, which had more Ce^3+^ species at the surface. The results show that temperature treatment of nanoceria can produce significant differences in solubility and surface cerium valence, which affect the biological and catalytic properties of nanoceria.

## Introduction

The long-term fate of engineered nanomaterials (ENMs), which could profoundly influence their biological effects, is not well understood. After uptake into phagolysosomes, which have a pH value of ca. 4.5, there is the potential for dissolution, changing the physicochemical, and potentially the biological, identity of ENMs.

Nanoceria are a family of metal oxide ENMs used industrially, as catalysts in diesel fuel, abrasives in chemical mechanical planarization, in integrated circuit manufacture, as structural supports for catalysts for fuel synthesis applications, in solid oxide fuel cells, and in rechargeable batteries [1–2]. Cerium oxide was selected by the OECD Working Party on Manufactured Nanomaterials as one of 13 representative manufactured nanomaterials for safety testing to create an understanding of the kind of information about intrinsic nanomaterial properties that may be relevant for exposure and effects assessment [3]. A nanoceria material, NM-212, produced by Umicore, has been studied to address the OECD objective.

Pulmonary NM-212 exposure for up to 90 days in rats resulted in pulmonary inflammation and genotoxicity with little evidence of clearance [4–8]. The European Union funded a comprehensive two-year whole-body combined chronic toxicity/carcinogenicity inhalation study in female rats exposed to 0, 0.1, 0.3, 1, or 3 mg/m^3^ NM-212, six hours daily on five consecutive working days per week (EU FP7 project ‘NANoREG’, Grant Agreement number 310584). Lung cerium burden after three, twelve, and 24 months of exposure positively correlated with exposure time and nanoceria concentration, with deposition fractions of 12%, 15%, 14%, and 8%, and predicted clearance half-lives of 86, 114, 164, and 200 days [9]. These studies showed that pulmonary exposure to NM-212 could lead to long-term biological persistence and have adverse consequences and raised the question whether its method of preparation and solubility are contributing factors.

In contrast to the results with NM-212, other nanoceria have been demonstrated to have therapeutic potential for multiple conditions with an oxidative stress/inflammation component, including cancer, radiation damage, bacterial infection, sepsis, wounds, stroke-induced ischemia, retinal degeneration, and neurodegenerative diseases [10]. Other reviews provide more information on nanoceria synthesis methods and the resulting physicochemical properties of the products as well as the beneficial biological effects and human diseases that could potentially be treated [11], and the physicochemical properties that mediate the effects of nanoceria, its biochemical properties, biosynthesis, and its major biomedical applications, including biosensors [12]. Additional applications are cited in the introduction of [13]. These beneficial results have been obtained with nanoceria prepared by precipitation or solvothermal synthesis.

There are many reports of nanoceria synthesis, producing a myriad of products with different physicochemical properties tailored to their application. NM-212 was prepared as a nanoceria representative of those used in industrial applications. Nanoceria prepared for industrial applications are often exposed to high temperature (calcined), which increases particle size and produces crystalline particles with a predominance of surface Ce^4+^ [14–18]. NM-212 has been reported to have a primary particle size of 20 to 40 nm and a predominance of surface Ce^4+^ (Supporting Information File 1, Table S1). In contrast, nanoceria demonstrated to have therapeutic potential are typically prepared by procedures that do not include calcination, resulting in primary particles ca. 5 nm in diameter and with a predominance of surface Ce^3+^ (Supporting Information File 1, Table S1). Nanoceria are autocatalytically redox-active, cycling between Ce^3+^ (anti-oxidative) and Ce^4+^ (pro-oxidative) species. A higher temperature during nanoceria preparation is generally associated with more nanoceria-induced pro-oxidative effects [19] and would be expected to produce a nanoceria form with more Ce^4+^ and less ability to autocatalytically cycle between Ce^4+^ and Ce^3+^.

Early reports stated that nanoceria were insoluble or did not demonstrate significant solubility (Supporting Information File 1, Table S2). However, when studied for sufficient time it has been shown that some nanoceria dissolve in acidic media, accelerated by carboxylic acids, including a ca. 5 nm solvothermally synthesized nanoceria prepared for biological assessment. Although there have been some investigations of NM-212 that found very low solubility, none investigated concurrent carboxylic acid exposure or addressed why the dissolution of NM-212 is so low. The rate and extent of nanoceria solubility can influence resultant effects, which are greater from the released cerium ions than from nanoceria [20–23]. Given that nanoceria prepared for industrial applications, such as NM-212, are typically calcined, it was hypothesized that the low solubility of NM-212 is a product of high-temperature exposure. NM-212 was prepared by precipitation [7,24], but the synthesis process has not been reported and could not be discovered by the authors. The JRC Nanotechnology team support was unable to provide synthesis procedure information (personal communication to R. Yokel, February 10, 2020). Given the diversity of nanoceria products with different physicochemical properties, which are influenced by their preparation procedures, this lack of information hinders the interpretation of the influence of the NM-212 preparation on its effects. This limitation also applies to reports of effects following pulmonary exposure to other commercially supplied nanoceria prepared by proprietary methods [25–32].

The objective of the present studies was to determine the long-term dissolution of NM-212 (as a representative nanoceria material prepared for industrial applications) compared to a solvothermally synthesized nanoceria material (demonstrated to have pharmaceutical applications), the influence of carboxylic acids and immobilized phosphate on their dissolution, the influence of nanoceria after partial dissolution on a selected biological effect, and the surface reactivity. Based on reports of very limited NM-212 dissolution and physicochemical differences between nanoceria synthesized for industrial and biological applications, the former often including calcination and producing larger particles, it was hypothesized that the long-term dissolution of NM-212 would be slower than that of a solvothermally synthesized nanoceria material. Given that cerium forms very stable complexes with phosphate but phosphate inhibits nanoceria dissolution, it was hypothesized that phosphates not in direct contact with nanoceria would create a sink for released cerium ions and increase the nanoceria dissolution rate. Assuming that NM-212 was calcined to a crystalline form with fewer oxygen vacancies and exhibits, therefore, a predominance of surface Ce^+4^, compared to a solvothermally synthesized nanoceria material with more oxygen vacancies, which produce a preponderance of surface Ce^+3^, it was hypothesized the NM-212 would have less catalytic activity.

Another nanoscale ceria, produced for OECD-suggested safety testing, NM-211, was included in some of the present studies for comparison to NM-212 and the solvothermally synthesized nanoceria material. Its size and percentage of surface Ce^+4^ are intermediate between NM-212 and the tested solvothermally synthesized nanoceria material [33].

Nanoceria dissolution was determined using two methods, a continuous flow system (CFS) and a dialysis system. The influence of immobilized phosphate on the nanoceria dissolution rate was determined by addition of two phosphate-containing resins to the dialysis system. The influence of nanoceria dissolution on its biological identity was assessed as the protein carbonyl level in response to partially dissolved nanoceria. The catalytic potential was assessed using a reactivity assay.

## Experimental

### Materials

NM-211 and NM-212 were obtained from the European Commission Joint Research Centre Institute for Health and Consumer Protection repository of reference materials from the OECD sponsorship program. The solvothermally synthesized nanoceria material was prepared using a hydrothermal method [34]. Briefly, 0.25 M cerium chloride heptahydrate and 0.25 M citric acid monohydrate were added to 1.5 M ammonium hydroxide, stirred, and autoclaved for 24 h at 50 °C then 24 h at 80 °C. The product was dialyzed against 110 mM pH 7.4 citric acid for 120 h, replacing the dialysate every 24 h, then dialyzed against DI water to remove citric acid and ionic cerium for an additional 72 h, replacing the dialysate every 24 h. The product was extensively characterized. It was a citrate-coated 4.2 nm ceria [35]. Lanthanides (LN) resin (100 to 150 μm) was obtained from Eichrom Technologies LLC, Lisle, IL, 60532. Hydroxyapatite (Bio-Gel HTP, 10 to 90 μm) was obtained from Bio-Rad. Cerium was obtained as an Aldrich ICP/DCP standard. RAW 264.7 (murine macrophage) cells were obtained from ATCC. Human blood serum was acquired from Sigma Aldrich (P2918-100 mL).

### Physicochemical characterization of NM-212

The NM-212 sample was physicochemically characterized to verify and expand on prior characterizations. Transmission electron microscopy (TEM) and high-angle annular dark-field scanning transmission electron microscopy (HAADF-STEM) were conducted of NM-212 dispersed in DI water, sonicated for 10 min in a bath, and captured on 300 mesh lacey carbon copper grids dipped into the dispersion for approximately 5 s and dried overnight at room temperature. Electron microscopy was performed on a Thermo Scientific Talos F200X operated at an accelerating voltage of 200 keV. Images were recorded with a Ceta CCD camera. The mean and Feret diameters and area, and their standard deviation, minimum, and maximum, of one hundred particles were calculated using ImageJ software. Energy-dispersive X-ray spectroscopy (EDS) and electron energy loss spectroscopy (EELS) were conducted using Thermo Scientific’s SuperX G2 and Gatans’ Enfinium ER, respectively. Thermogravimetric analysis (TGA) (PerkinElmer TGA7) was used to determine the organic weight percent. This was repeated three times. TGA runs were completed under a nitrogen atmosphere to prevent the oxidation of organic matter. The sample was heated from 20 to 125 °C at 10 °C/min, held at 125 °C for 30 min to release physiosorbed water, and then heated to 900 °C at 10 °C/min. Fourier-transform infrared spectroscopy (FTIR) (Nicolet 6700 FTIR with a diamond ATR crystal) was used to detect organic functional groups on the ENM surface. Thirty-two scans were completed.

### Nanoceria calcination

Two samples (6.5 and 6.7 mg) of solvothermally synthesized nanoceria, described in Supporting Information File 1, Table S1, were calcined by heating in porcelain crucibles in a muffle furnace to 900 °C at a ramp rate of ca. 10 °C/min, then held for 3 h. The product was characterized by electron microscopy performed on the Thermo Scientific Talos F200X described above. The primary particle diameter was estimated from 100 particles.

### Nanoceria dissolution

The continuous flow system has been described [36–37]. It consists of phagolysosomal simulant fluid (Aleks Stefaniak’s PSF medium at pH 4.5 [38]), the flow-through cell, a flow regulating pump, and an autosampler. In the PSF reservoir, ultrahigh molecular weight polyethylene (UHMWPE) filters prevent clogging of the ultrafiltration membrane in the flow cell. For the standard conditions, 1 mg of ENM was placed on a 5,000 MWCO, 47 mm cellulose triacetate membrane (Stedim Biotech GmbH, Göttingen, Germany, 14529-47-D) and inserted into the flow-through cell (BOLA, Bohlender GmbH, Germany, N1682-08). Over seven days, the ENM was exposed to a continuous 2 mL/h PSF flow at 37 °C. During this time, ten samples were drawn, and the eluate subsequently analyzed for its elemental concentration as described in section “Cerium quantification”.

The dialysis system has been described [39–40]. Nanoceria (500 μg cerium as nanoceria in 1 mL) was loaded into Slide-A-Lyzer^TM^ dialysis cassettes with 2 kD MWCO regenerated cellulose membranes (66203) immersed in a 200 mL bath at pH 4.5 made iso-osmotic with sodium nitrate. Cassette/beaker systems were housed in a rotary shaking incubator at 37 °C rotated at 60 rpm. Dissolution was determined from the bath cerium concentration measured weekly for 2688 h (16 weeks). Citric and lactic acids (110 mM in the baths), previously shown to accelerate nanoceria dissolution [39–40], were studied. All conditions were studied in duplicate. To test the hypothesis that the slower dissolution of NM-212 was due to elevated temperature exposure during its preparation, the dissolution of calcined samples of the solvothermally synthesized nanoceria was compared to the non-calcined form.

### Nanoceria dissolution in the presence of immobilized phosphates

Two immobilized phosphates were studied to assess their effect on the dissolution of solvothermally synthesized nanoceria. NM-212 was not studied as it did not appreciably dissolve. LN resin, developed for extraction chromatography, containing di(2-ethylhexyl) orthophosphoric acid as the metal complexing group, and hydroxyapatite (HTP) were studied. Initial experiments were conducted to verify their ability and capacity to complex ionic cerium. LN resin (7.5, 40, or 75 mg) was added to duplicate 15 mL tubes containing 10 mL pH 4.5 or pH 7 iso-osmotic citrate and 500 μg ionic cerium. The tubes were gently agitated at 37 °C and aliquots of 75 μL were withdrawn after 0, 0.5, 1, 2, 4, 6, and 24 h for cerium quantitation. The results showed that 40 and 75 mg LN resin complexed more than 50% of the cerium ions within 0.5 h at pH 4.5. This experiment was repeated with 40 or 75 mg LN resin and sampling up to 3360 h. This experiment was similarly conducted with 75 mg HTP at pH 4.5 and pH 7 with samples collected up to 2016 h.

To test the hypothesis that phosphates not in direct contact with nanoceria increase the nanoceria dissolution rate, LN resin and HTP were added to the dialysis system bath (75 mg, LN at pH 4.5 and HTP at pH 7.4). The cassette contained 500 μg cerium (as solvothermally synthesized nanoceria in 1 mL). In an initial experiment, samples were withdrawn from duplicate cassettes five times from 1344 to 4704 h. The cerium concentration in the cassettes decreased much faster at pH 4.5 than at pH 7.4, consistent with prior results [39–40]. The experiment was repeated with additional duplicate systems containing no LN resin at pH 4.5 and no HTP at pH 7.4. Cassette samples were withdrawn weekly for 16 weeks (from 168 to 2688 h).

### Cerium quantification

Samples were digested with 2:1 HNO_3_/H_2_O_2_ in Teflon vessels in a CEM MARS Express microwave digestion system. Terbium was added as an internal standard. Samples were analyzed compared to standards. At the University of Kentucky, cerium was quantified by inductively coupled plasma mass spectrometry (ICP-MS; Agilent 7500cx or 7900, Agilent Technologies, Inc., Santa Clarita, CA) [41]. Spiked samples (cerium average recovery was 106%) and blank samples (that were below the detection limit) were included. At BASF, the eluate was diluted 10- to 100-fold and quantified by ICP-MS (Perkin Elmer Nexion 2000b, Perkin Elmer Inc., Waltham, USA) with a limit of detection of 0.01 ppb. Measurements were conducted in triplicate and averaged.

### Determination of dissolution half-life and dissolution rate

The dissolution half-life was calculated as described [37,42] as a curve fitting of the cumulative dissolved mass, expressed as an inverse relationship of decreasing solid retained (CeO_2_ mass) as (*M*_ion_(*T*) − *M*_0_)/*M*_0_, where *M*_ion_ is the amount of dissolved nanoceria and *M*_0_ is the initial mass loading, and plotted as a function of the time on a semi-log scale. The dissolution rate (*k*) was determined from the slope of the line as shown below, normalized to the surface area determined using the Brunauer–Emmett–Teller method, *A*_BET_, and converted to percent per day of *M*_0_. Dissolution rate and half-life (*t*_1/2_, 50% dissolved) are inversely related and can be expressed as given for first-order modeling in ISO 19057:2017 [43–44]:


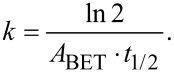


### Biological identity of the as-prepared and partially dissolved solvothermally synthesized nanoceria

Solvothermally synthesized nanoceria was partially dissolved by loading nanoceria (1.3 mL of 19,540 μg/mL cerium) into Slide-A-Lyzer^TM^ cassettes immersed in 200 mL of pH 4.5 iso-osmotic citric acid, and agitated as described above in section “Nanoceria dissolution”. Samples withdrawn from a cassette after 75, 102, and 152 days were dialyzed against water, as described above in the synthesis of the solvothermally synthesized nanoceria. The hydrodynamic diameter (Smoluchowski's approximation) of the as-prepared- (as-loaded) and the partially dissolved nanoceria was determined by dynamic light scattering (DLS) using a Brookhaven 90Plus Particle Size Analyzer. The zeta potential (0.5 mg/mL) from pH 0.5 to 13, adjusted with nitric acid and sodium hydroxide, was determined using an Anton Paar Litesizer 500 Particle Analyzer. The instrument was equipped with a 40 mW laser emitting at 658 nm. One hundred runs were completed in sequence with a 30 s equilibration time at 25 °C. Based on the bath cerium concentration after 75, 102, and 152 days the nanoceria was dissolved to 38%, 47%, and 93%, respectively. In an initial experiment, RAW 264.7 cells were exposed for 24 h to 0, 1, 3, 10, 30, or 100 μg/mL of the 75 day partially dissolved nanoceria. The cells were washed twice with Dulbecco’s PBS, pelleted, and stored at −74 °C. Cells were homogenized in the presence of protease inhibitors (4 µg/mL leupeptin, 4 µg/mL pepstatin A, and 5 µg/mL aprotinin). Protein was measured using the bicinchoninic acid method. Protein carbonyls were determined as described in [45]. Briefly, samples were derivatized with 10 mM 2,4-dinitrophenylhydrazine solution in 12% sodium dodecyl sulfate for 20 min at room temperature, neutralized, and blotted onto a nitrocellulose membrane for slot-blot analysis with an anti-DNPH rabbit antibody and secondary anti-rabbit IgG alkaline phosphatase antibody, using reagents from the OxyBlot Protein Oxidation Detection Kit (Millipore). The initial experiment was repeated with as-prepared and 75, 102, and 152 day partially dissolved nanoceria.

### Nanoceria reactivity

Oxidative stress, as mass-based biological oxidative damage (mBOD), and surface-based biological oxidative damage (sBOD), of NM-211, NM-212, and the solvothermally synthesized nanoceria was determined using the ferric reduction ability of serum assay (FRAS) as described in [46]. The materials were tested in triplicate, at a concentration of 1 m^2^ ENM/mL serum.

## Results

### Physicochemical characterization of NM-212

**Figure 1:**
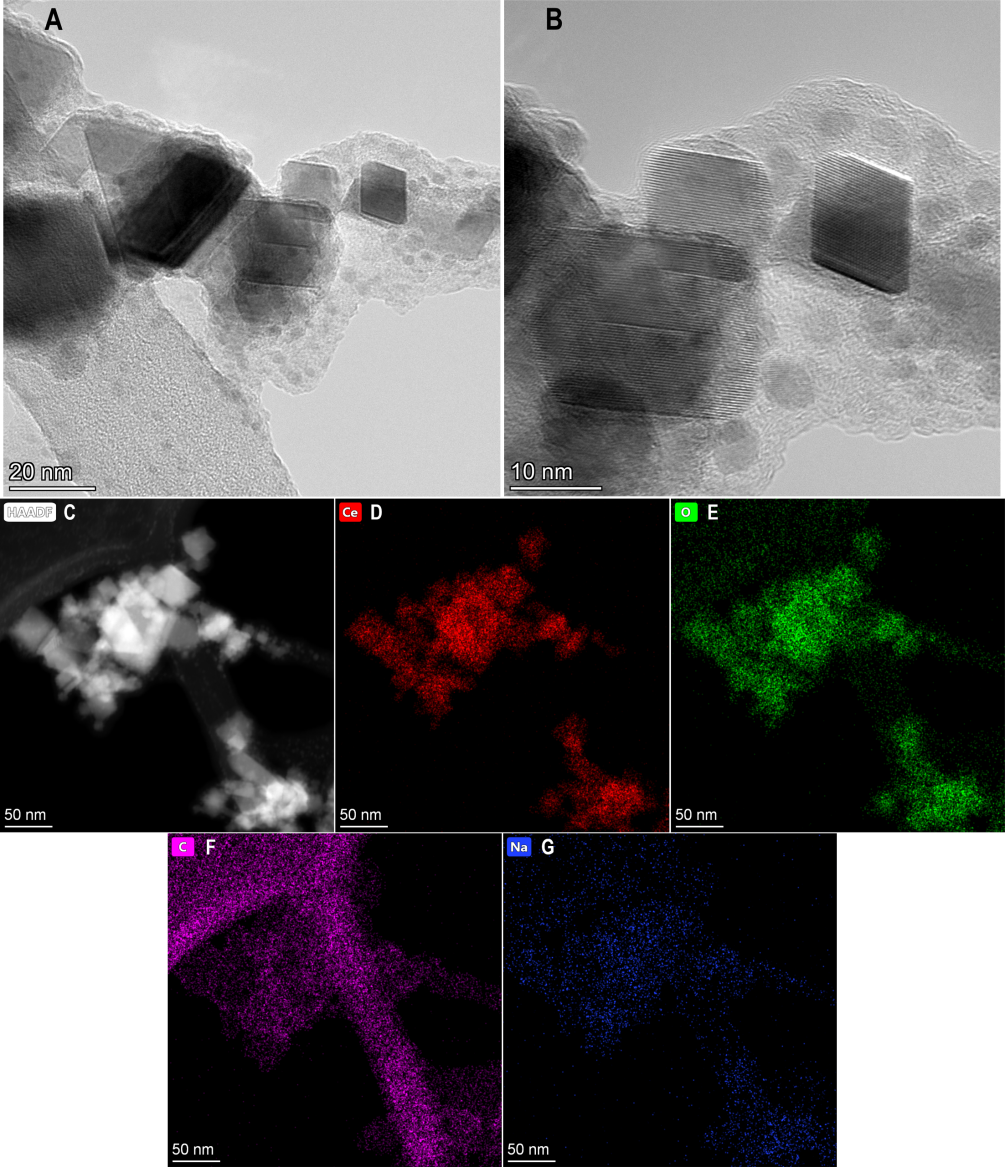
Transmission electron microscopy and high-angle annular dark-field scanning transmission electron microscopy images of NM-212 and its chemical composition determined by energy-dispersive X-ray spectroscopy. (A, B) TEM images, (B) is a part of (A) at higher magnification. (C) HAADF STEM image. (D–G) EDS-determined chemical composition.

**Figure 2:**
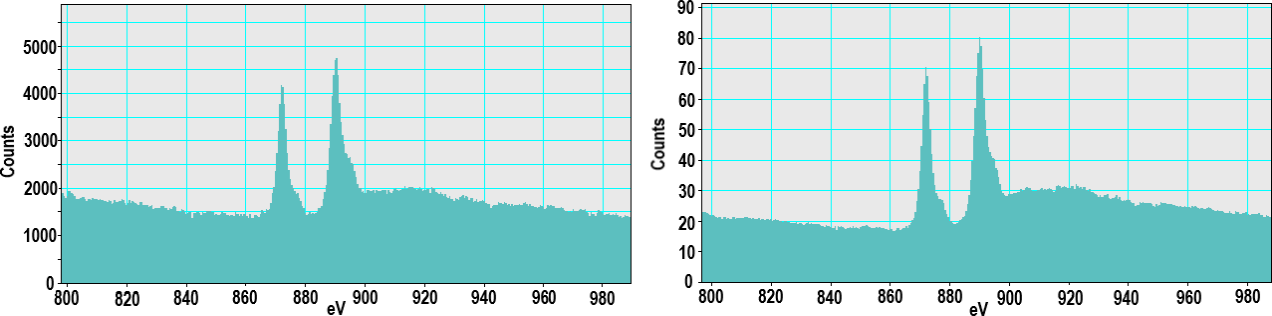
NM-212 cerium valence determined by electron energy loss spectroscopy. The left image was obtained at the edge, the right image at the ENM core.

**Figure 3:**
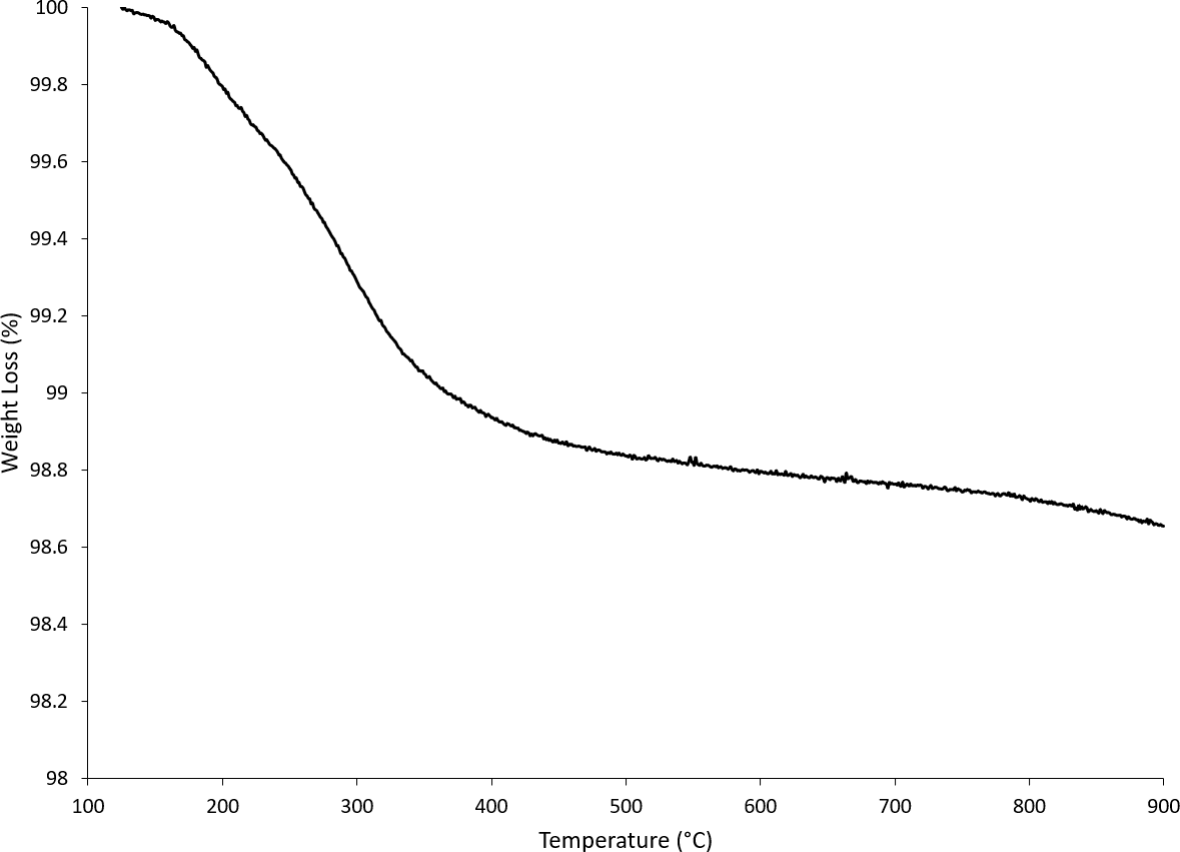
Thermogravimetric analysis of NM-212. Representative and near average of three determinations.

**Figure 4:**
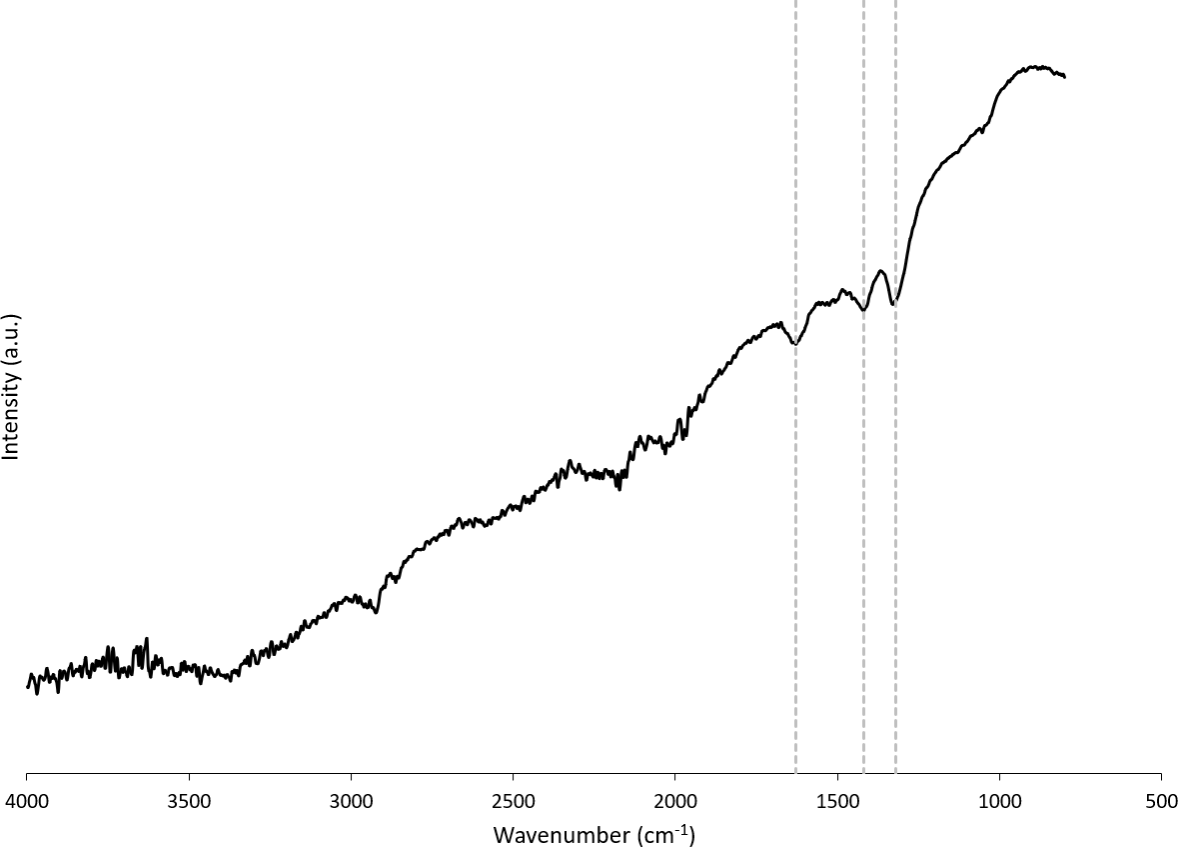
Non-baseline corrected Fourier-transform infrared spectroscopy of NM-212 with peaks indicated by dashed lines at 1630, 1420, and 1320 cm^−1^.

TEM, conducted at a higher resolution than previously reported, showed NM-212 to be crystalline and cubic or triangular with clearly defined edges (Figure 1). The solvothermally synthesized nanoceria is also crystalline but hexagonal and smaller (Figure 1b of [35]). The estimated diameter and variability of NM-212 (Table 1) are consistent with prior reports (Supporting Information File 1, Table S1) and the size is consistent with nanoceria calcined at temperatures above 600 °C [15–17]. The relative intensities of the M5 and M4 peaks are directly related to the Ce^3+^ and Ce^4+^ concentrations, respectively [47–48]. EELS showed that the NM-212 particle edge and core exhibit ca. 90% Ce^4+^ (Figure 2), consistent with nanoceria calcined at *T* ≥ 400 °C [18]. In contrast, the surface of the solvothermally synthesized nanoceria exhibits primarily Ce^3+^ (Figure 3b of [35]). TGA showed an average weight loss from 20 to 400 °C of ca. 1.3% for NM-212 (Figure 3), indicative of some organic surface coating, as previously reported [7]. The TGA weight loss for the solvothermally synthesized nanoceria was much greater (ca. 15%) due to its citrate coating (Figure 8 of [35]). FTIR, not previously reported for NM-212, showed small peaks at ca. 1630, 1420, and 1320 cm^−1^, attributed to N–O, –COOH, or hydroxy groups; probably COOH and the stretching mode of O–H bonds; and either –CH or C–O–C, respectively [16,49–50] (Figure 4). Due to the citrate coating on the solvothermally synthesized nanoceria, the FTIR spectrum shows additional large peaks at 1535 and 1365 cm^−1^ (Figure 10 of [35]). EDS showed co-localization of not only cerium and oxygen for both NM-212 (Figure 1) and the solvothermally synthesized nanoceria (Figure 2 of [35]), but also carbon, as previously reported [7], and sodium (Figure 1) were found.

**Table 1:** NM-212 diameter and area determined from TEM images.

	estimated diameter (nm)	Feret diameter (nm)	area (nm^2^)

mean	22	29	484
S.D.	11	15	490
minimum	8	11	55
maximum	59	85	2775

### Calcined nanoceria physicochemical characterization

**Figure 5:**
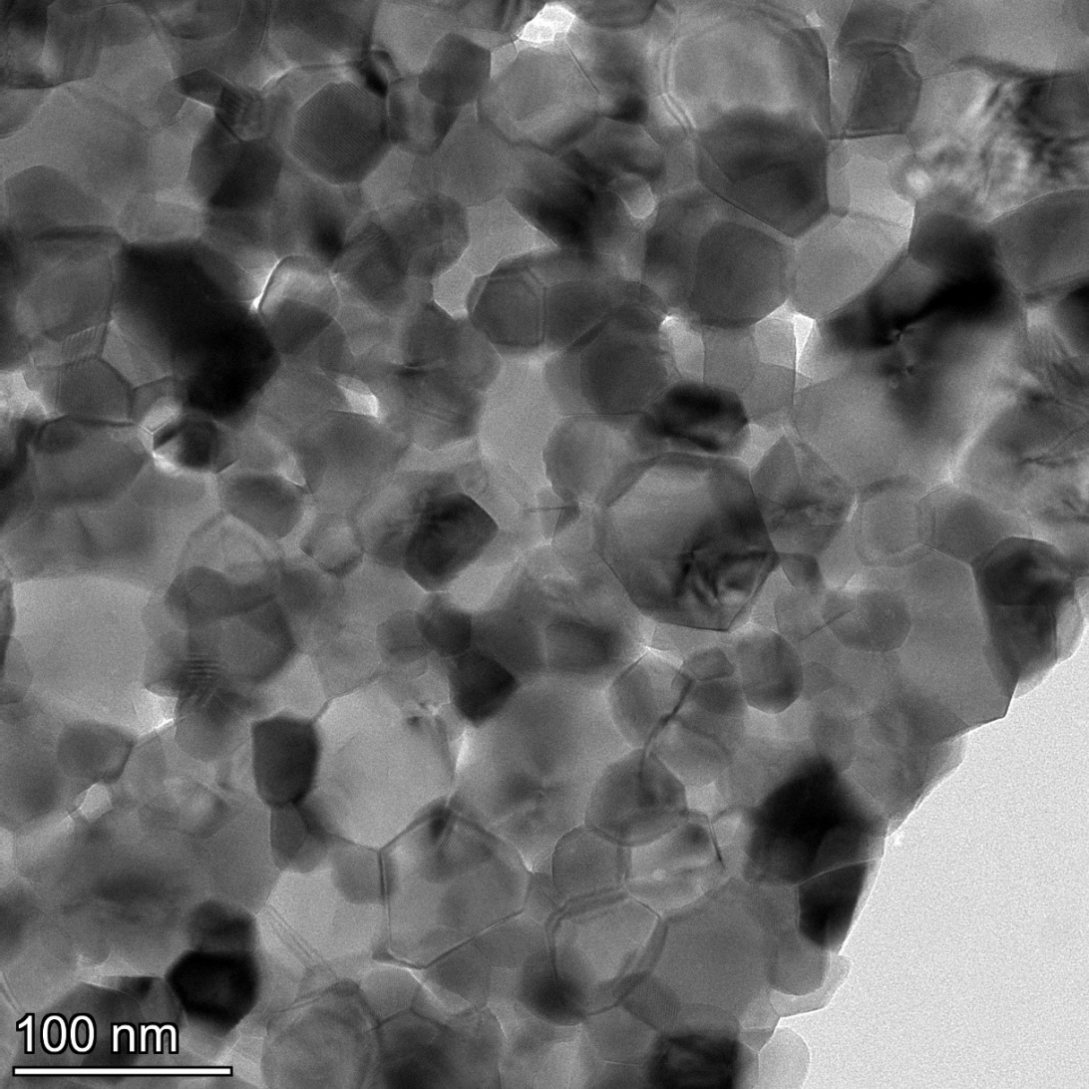
Transmission electron microscopy image of calcined solvothermally synthesized nanoceria.

**Figure 6:**
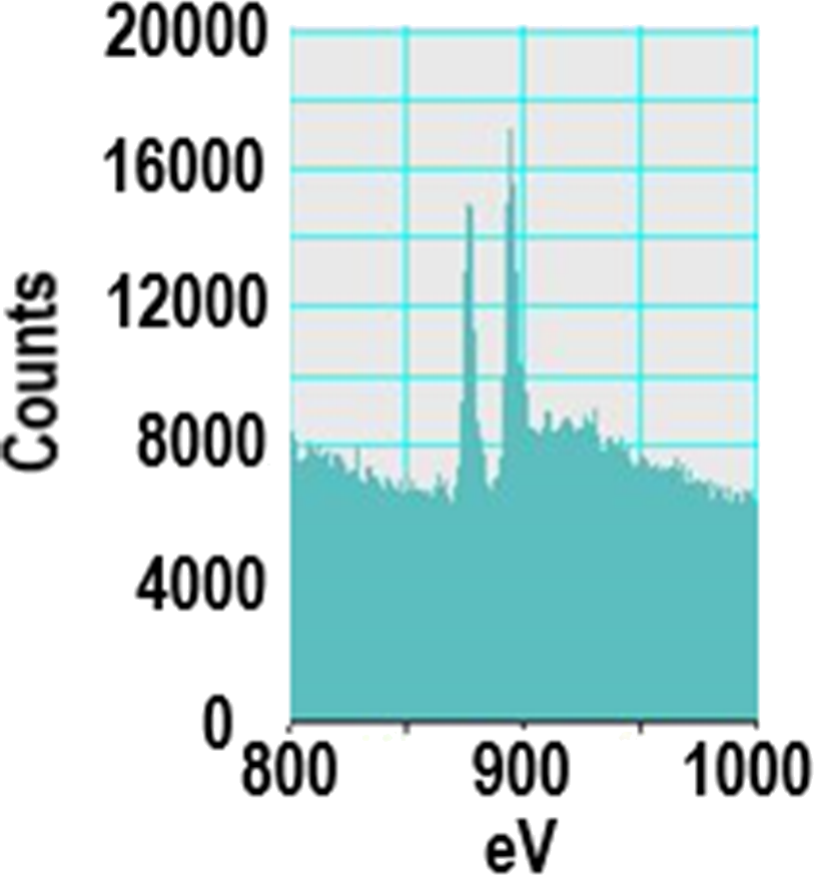
Electron energy loss spectroscopy of calcined solvothermally synthesized nanoceria.

TEM showed the calcined nanoceria was crystalline with a primary particle diameter of 41 ± 11 nm (mean ± S.D.) and had formed large aggregates (Figure 5). EELS showed a predominance of Ce^4+^ (Figure 6). HAADF-EDS confirmed the presence of cerium and oxygen and showed some nitrogen and sodium but no carbon above the background.

### Nanoceria dissolution

**Figure 7:**
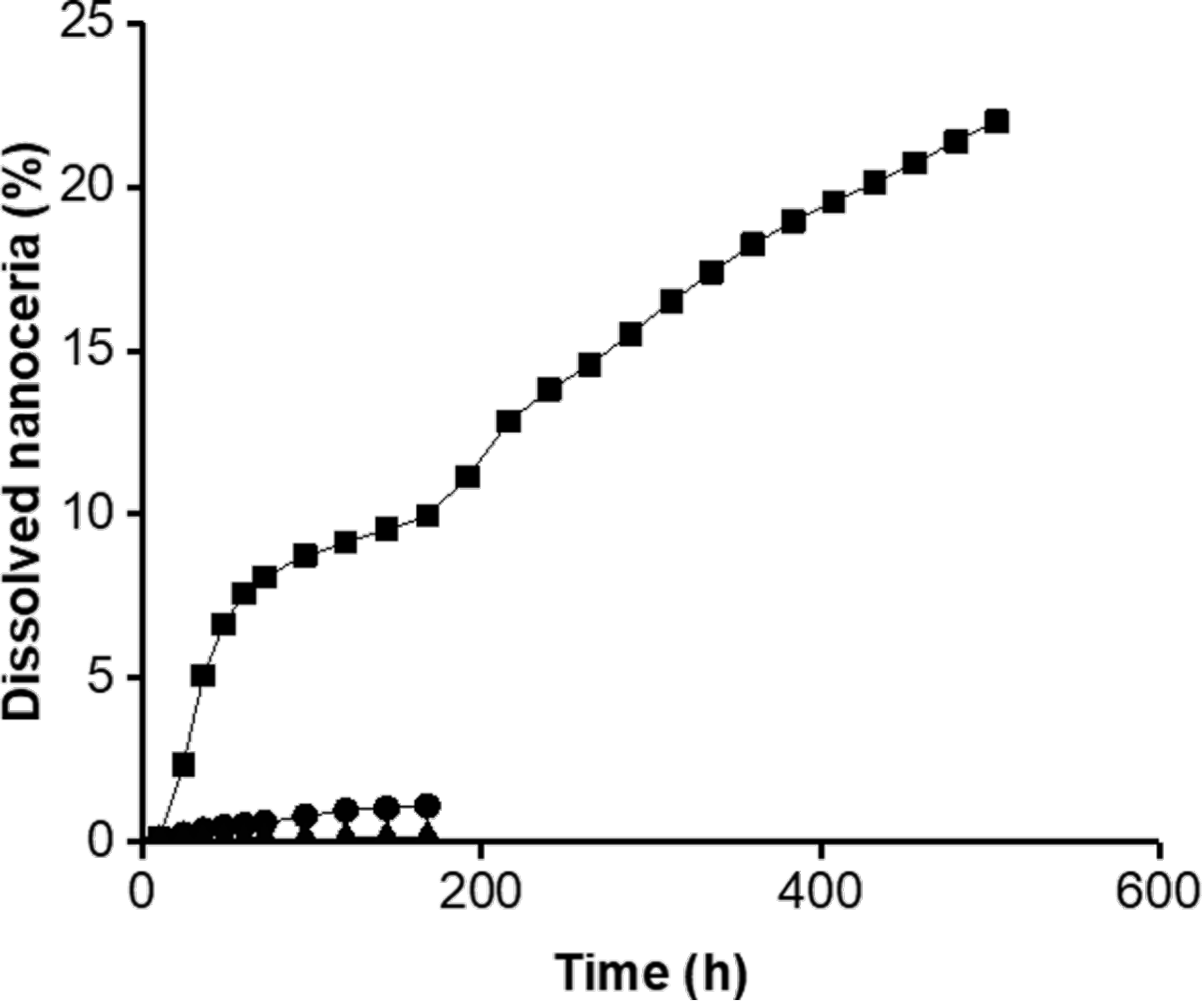
Dissolution of NM-211 (circles), NM-212 (triangles), and the solvothermally synthesized nanoceria (squares) determined in a single experiment using the continuous flow system.

Under identical conditions of continuous flow testing at pH 4.5, the solvothermally synthesized nanoceria dissolved significantly faster than NM-212 or NM-211 (Figure 7), calculated from ICP-MS determination of dissolved ions. The difference between NM-212 and the solvothermally synthesized nanoceria is less dramatic if the results are expressed as a dissolution rate in which the difference in specific surface area is factored out. Assuming first-order kinetics, as successfully fit to other, more soluble, ENMs [36], the half-lives of the solvothermally synthesized nanoceria and of NM-212 are ca. 50 and ca. 2250 days, respectively (Table 2).

**Table 2:** Half-lives and dissolution rates of the tested nanoceria.

		half-life [d]	dissolution rate [atoms/(h·nm^2^)]

continuous flow system			

solvothermally synthesized nanoceria	PSF pH 4.5	46.8	0.014
NM-211		513	0.0036
NM-212		2263	0.0020

dialysis system			

solvothermally synthesized nanoceria	no carboxylic acid	—	0.010
	citric acid	—	0.015
	lactic acid	—	0.014
	absence of LN pH 4.5	—	0.018
	presence of LN pH 4.5	—	0.017
	absence of HTP pH 7.4	—	0.0090
	presence of HTP pH 7.4	—	0.0078
NM-212	no carboxylic acid	—	0.00066
	citric acid	—	0.0012
	lactic acid	—	0.0012

**Figure 8:**
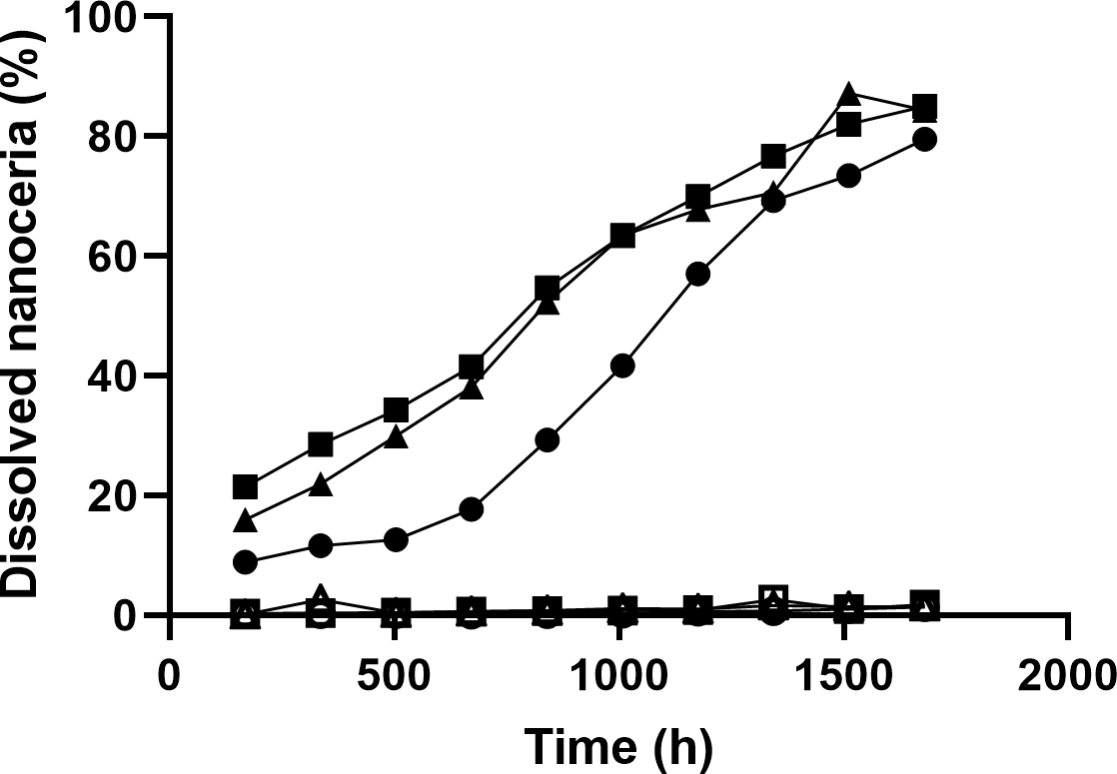
Dissolution of NM-212 and the solvothermally synthesized nanoceria, in the absence and presence of citric and lactic acids, determined by the dialysis system. NM-212 in the absence and presence of citric and lactic acids are indicated by open circles, squares, and triangles, respectively. Solvothermally synthesized nanoceria in the absence and presence of citric and lactic acids are indicated by solid circles, squares, and triangles, respectively. Results are the mean of two observations.

**Figure 9:**
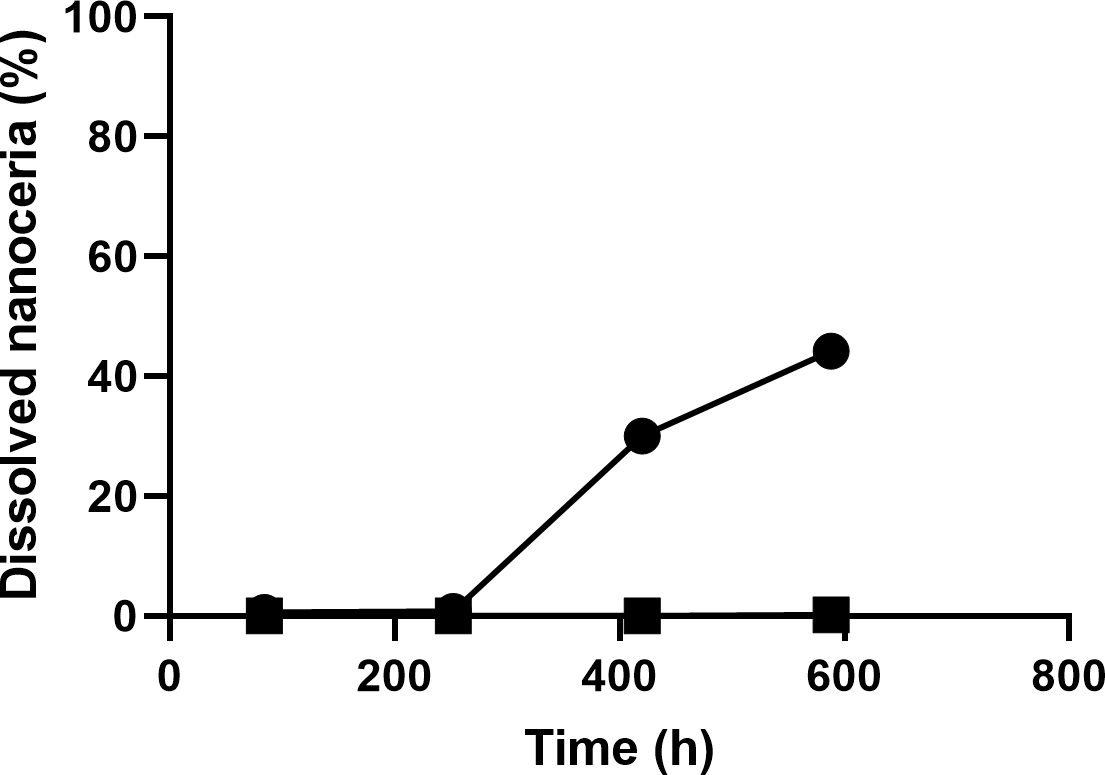
High-temperature exposure decreased the dissolution of solvothermally synthesized nanoceria. Solid circles represent solvothermally synthesized nanoceria (non-calcined) and squares the calcined form. Results are the mean of two observations.

The cerium concentration in the bath of the dialysis system that contained solvothermally synthesized nanoceria in the cassette increased over time, indicating nanoceria dissolution. Citric and lactic acid increased the dissolution rate (Table 2), as previously reported [39–40]. The rate of NM-212 dissolution was much slower (Figure 8 and Table 2). High-temperature exposure greatly decreased the dissolution of the solvothermally synthesized nanoceria (Figure 9).

### Cerium ion complexation and nanoceria dissolution in the presence of immobilized phosphates

**Figure 10:**
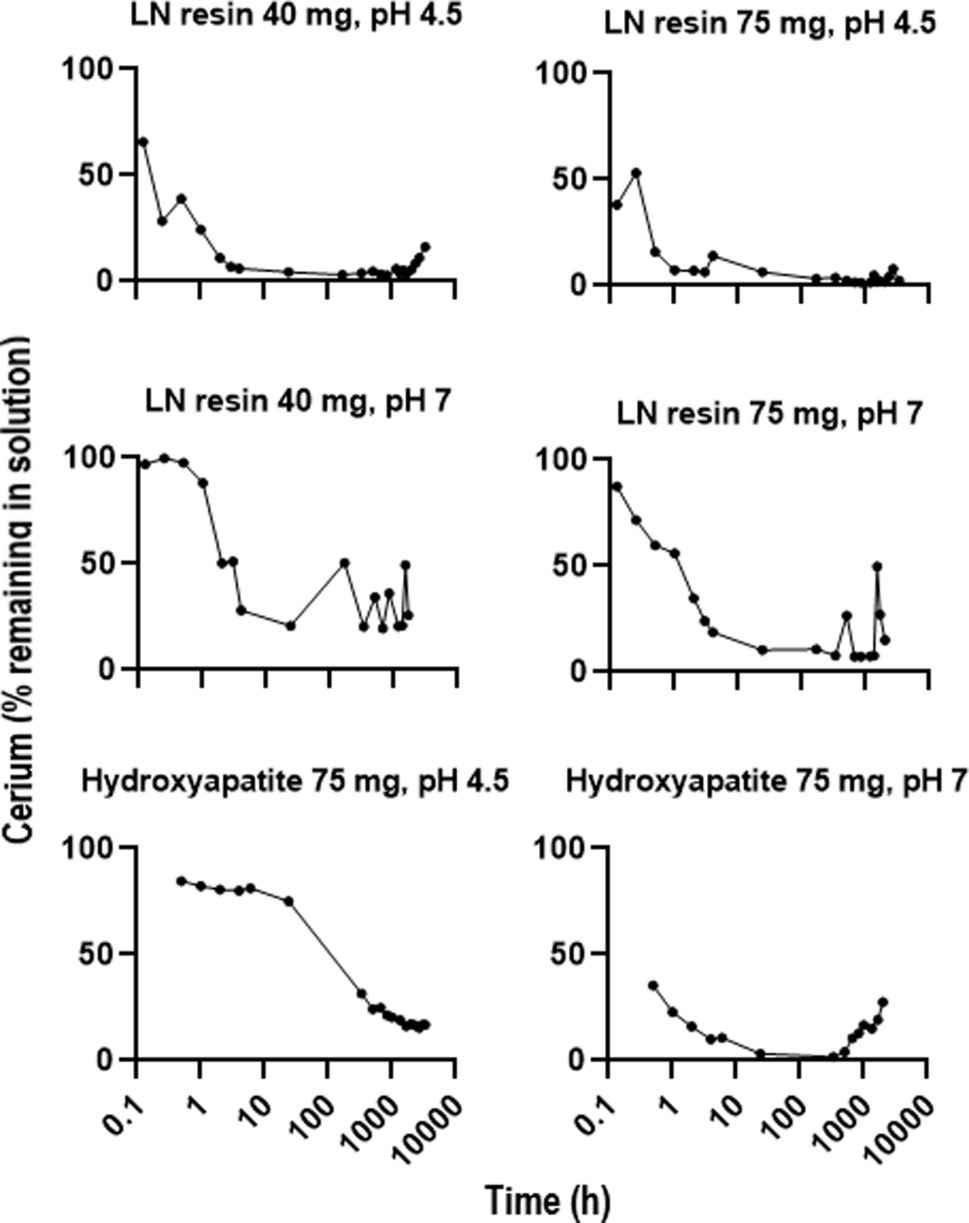
Dependency on the pH value of the complexation of cerium ions with LN resin and HPT. Results are the mean of two observations.

**Figure 11:**
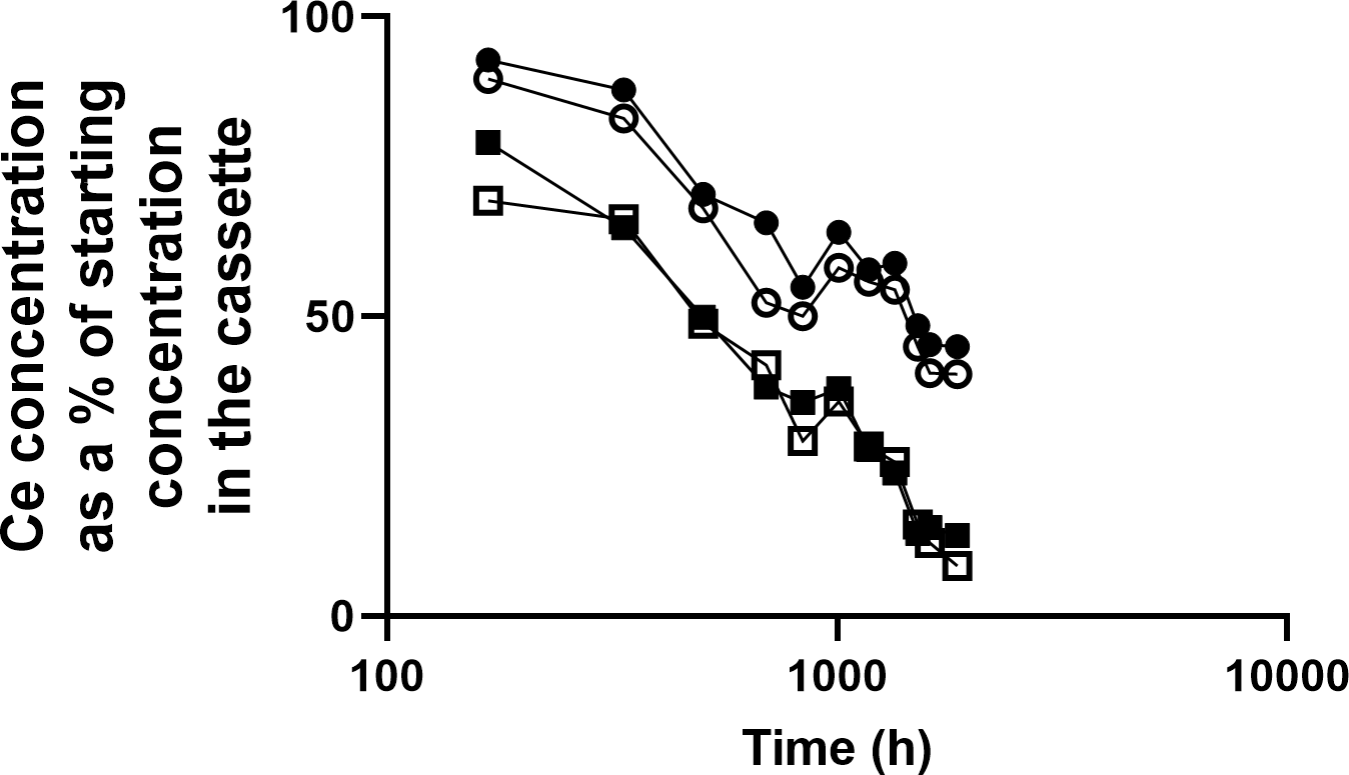
Addition of LN resin and HTP to the dialysis bath did not affect nanoceria dissolution. Solid circles: with HTP at pH 7.4, open circles: without HTP at pH 7.4, solid squares: with LN resin at pH 4.5, open squares: without LN resin at pH 4.5. Results are the mean of two observations.

The LN resin complexed more cerium ions from the solution, and more rapidly, at pH 4.5 than at pH 7 (Figure 10). After ca. 2000 h at pH 4.5, some cerium started to be released from the LN resin. HTP more efficiently complexed cerium ions at pH 7 than at pH 4.5 (Figure 10). After ca. 500 h at pH 7, some cerium started to be released from HTP. When LN resin or HTP was added to the bath of the dialysis system containing cassettes with nanoceria, the decline rate of the cerium concentration in the cassette did not differ from systems not containing resins (Figure 11 and Table 2). The nanoceria mean residence time from time zero to the time of the last measured concentration (MRT_last_), calculated by Phoenix 8.1 WinNonLin, in the absence and presence of the LN resin at pH 4.5 was 621 and 625 h, respectively, and in the absence and presence of HTP at pH 7.4, it was 786 and 797 h, respectively.

### Biological identity of the as-prepared and partially dissolved solvothermally synthesized nanoceria

The hydrodynamic diameter (as surface area) of the nanoceria materials after 75, 102, and 152 days of dissolution was 18 to 34 nm, 18 to 27 nm, and more than 1000 nm, respectively, compared to 6.5 to 13.2 nm for the as-prepared nanoceria. After 75 days of dissolution, the surface charge was −9 mV, compared to the as-prepared nanoceria surface charge of −40 mV, suggesting the loss of the citrate coating.

**Figure 12:**
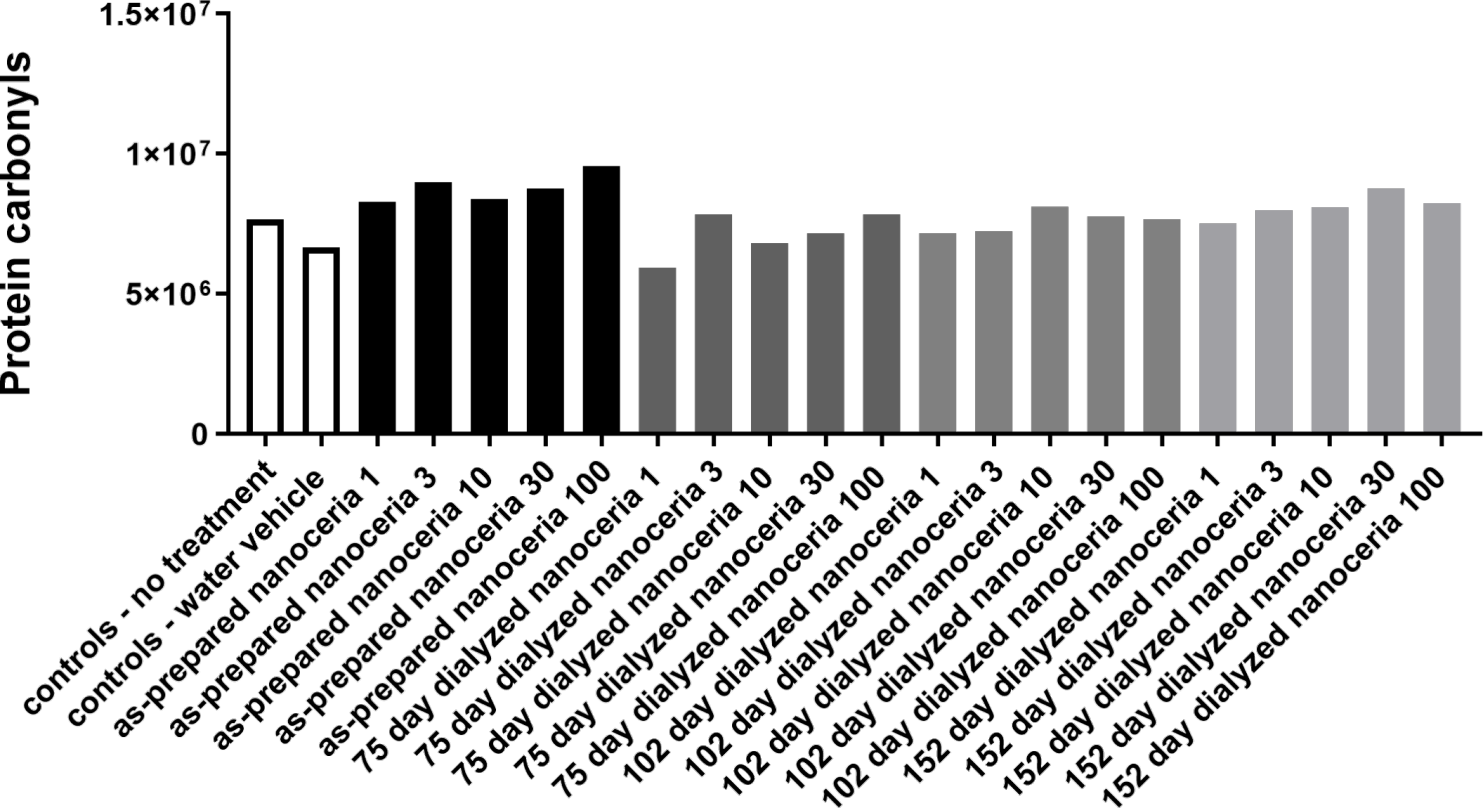
As-prepared and partially dissolved nanoceria altered cell protein carbonyl production. RAW 264.7 cell protein carbonyl production in response to the as-prepared and partially dissolved nanoceria. *X*-axis numbers refer to the nanoceria concentration in μg/mL. Results are the mean of two determinations from one experiment.

RAW 264.7 cell exposure to the as-prepared solvothermally synthesized nanoceria showed a trend toward higher protein carbonyl production compared to cells not exposed to nanoceria. The initial experiment of cells exposed to nanoceria that had been partially dissolved for 75 days showed a trend toward lower protein carbonyl production than that of cells exposed to the as-prepared nanoceria (results not shown). When this experiment was repeated, the as-prepared nanoceria increased the production of protein carbonyls. Nanoceria partially dissolved after 75, 102, and 152 days increased the production protein carbonyls to a lesser extent than as-prepared nanoceria (Figure 12).

### Nanoceria reactivity

The mBOD and sBOD values for NM-211, NM-212, and the solvothermally synthesized nanoceria are reported in Table 3 with information used to interpret the differential solubility and reactivity. The solvothermally synthesized nanoceria exhibited the highest reactivity with little difference between NM-211 and NM-212.

**Table 3:** Production process, producer, physicochemical properties, and reactivity of NM-211, NM-212, and the solvothermally synthesized nanoceria.

name	NM-211	NM-212	solvothermally synthesized nanoceria

production process	precipitated	precipitated	solvothermal
producer	JRC (Antaria)	JRC (Umicore)	University of Kentucky
crystallinity	cubic (cerianite) [7]	cubic (cerianite) [7,24,51]	hexagonal (cubic fluorite) [35,52]
surface treatment	none	none	citrate [35]
impurity (%)	ICP-MS: Al 0.2%, 19 other elements < 0.02% [24]	ICP-MS: Al 0.2%, 19 other elements < 0.02% [24]	
surface Ce^3+^ oxidation state (XPS)	5.7% [24]	6.9% [24]	predominantly Ce^3+^ [35,39,52]
primary size (D_50_) (nm)	15 [33]	40 [7,33]	4.2 [35,39,52]
specific surface area (m^2^/g)	66 [33]	27 [4–5,7,24,33,36,51]	197 (calculated from TEM)
dissolution rate in PSF (atoms/(h·nm^2^))	0.00365	0.00202	0.0144 (monomodal)
FRAS sBOD reactivity (dose 1 m^2^/mL) (nmol TEU/m^2^ ENM)	14 ± 1	13 ± 1	38 ± 5 (@ 0.3 m^2^/mL)
FRAS mBOD reactivity (nmol TEU/mg ENM)	0.9 ± 0.04	0.3 ± 0.02	7.5 ± 1.04

## Discussion

### Comparison of the physicochemical properties

TEM images obtained for this study suggest that NM-212 is crystalline and cubic or triangular with clearly defined edges (Figure 1). The solvothermally synthesized nanoceria is also crystalline but hexagonal. A previous determination of the primary particle size of NM-212 yielded an average of 20 to 40 nm with a very wide size range (Supporting Information File 1, Table S1). The size determination in this study showed an average diameter of 22 nm with a range of 11 to 59 nm. In contrast, the solvothermally synthesized nanoceria had a primary particle diameter of 4 nm. A prior characterization of the surface oxidation state of NM-212 showed a great predominance of Ce^4+^ (Supporting Information File 1, Table S1) which was also found in the present study at the edge and core of the particles (Figure 2). In contrast, the solvothermally synthesized nanoceria cerium surface valence was predominantly Ce^3+^ (Supporting Information File 1, Table S1), consistent with the increase in Ce^3+^ as the size of nanoceria decreases [53]. The calcined solvothermally synthesized nanoceria cerium had a predominance of Ce^4+^ (Figure 6). NM-212 was reported to be uncoated [24]. However, it was found that its surface had organic contaminations, shown by thermogravimetric analysis (<0.7 % of the material [7]). Further investigation with XPS, which has an information depth between 3 and 10 nm, indicated 80% carbon atoms on the surface. Photoelectron energies suggested the contamination could be an ester with a long alkyl chain. The contamination was a very thin, homogeneous layer [7]. TGA conducted in the present study showed an average weight loss of 1.3% (Figure 3) which can be attributed to a small amount of surface contamination. The comparison with the TGA-induced 5% weight loss of the solvothermally synthesized nanoceria at 125 °C due to outgassing of water, including surface hydroxy groups, suggests that NM-212 was calcined. The solvothermally synthesized nanoceria also exhibited a weight loss of ca. 10% due to its citrate coating. EDS revealed some carbon and sodium on the particle surface of NM-212 (Figure 1). FTIR showed small peaks at 1630, 1420, and 1320 cm^−1^, attributed to N–O or –COOH, probably –COOH, and either –CH or C–O–C, respectively, which would be consistent with the presence of an ester and an alkyl chain (Figure 4). In contrast, solvothermally synthesized nanoceria was coated with, on average, a monolayer of citrate, intentionally applied to inhibit agglomeration [35]. It is assumed that the citrate coating was dissolved as surface cerium ions were solubilized. The reduction in absolute zeta potential of the partially dissolved nanoceria supports this assumption.

**Figure 13:**
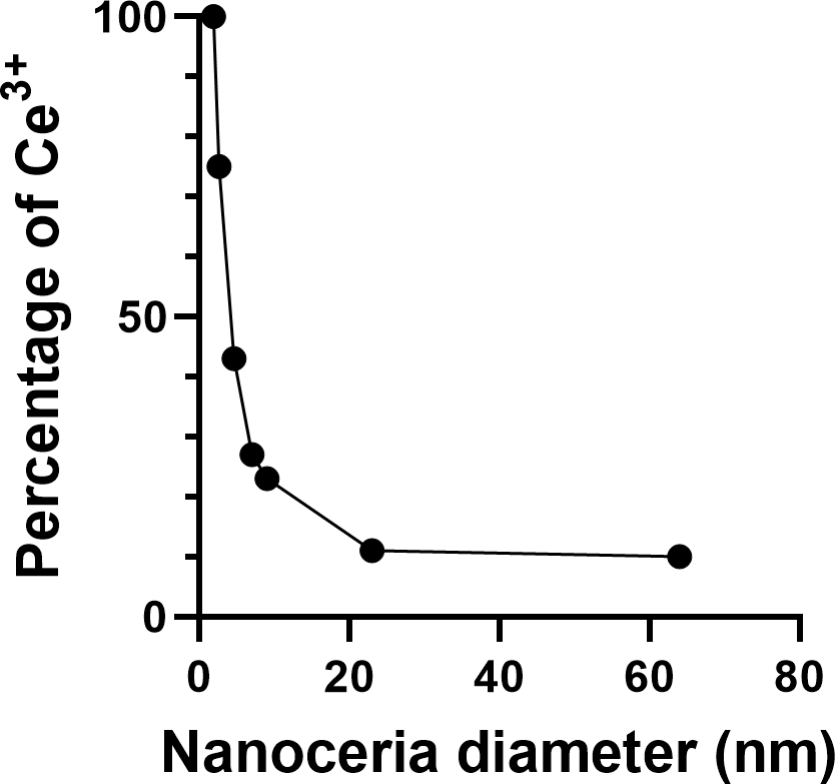
The percentage of surface cerium as Ce^3+^ as a function of the nanoceria size. Data taken from [55] and [56].

The high fraction of Ce^4+^ in the calcined solvothermally synthesized nanoceria is comparable to that of NM-212 and expected after calcination [18]. The increased primary particle size is consistent with reports that found a temperature-dependent increase of nanoceria particle size, including 44 nm particles when heated to 1000 °C [15–17]. The predominance of Ce^3+^ on the surface of the non-calcined solvothermally synthesized nanoceria is consistent with its small size [54] and Figure 13.

### Dissolution

Prior studies reported a solubility of less than 3% of NM-212 up to 28 d (672 h) in multiple media (see Introduction). The present results extend the dissolution time to 16 weeks (2688 h). Also, they include the addition of carboxylic acids that have been shown to accelerate nanoceria dissolution (Figure 8 and Table 2) and a direct comparison of the solubility of NM-212 to a commonly used solvothermally synthesized nanoceria by two methods conducted by different research teams. After 16 weeks, ca. 1.5% of the cerium in NM-212 was in the dialysis system bath, compared to ca. 80% from the solvothermally synthesized nanoceria. Several factors could contribute to the much more rapid dissolution of the solvothermally synthesized nanoceria. The size difference may contribute to the dissolution difference. The solvothermally synthesized nanoceria is smaller than NM-212 or the calcined solvothermally synthesized nanoceria (4.2, 40, and 41 nm diameter, respectively). The dissolution half-time of an ENM is inversely proportional to its surface area [40]. Concerning comparable masses, as introduced into the dissolution systems in this study, smaller ENMs would be expected to dissolve faster. But size alone does not account for the differences, because the difference remains very significant in surface-normalized dissolution rates. The specific surface area was factored out in the calculation of the dissolution rates from the half-lives, as is routine practice for the CFS results and given in [43].

The maximum temperature that the nanoceria materials were exposed to during preparation may contribute to the dissolution rate difference. Industrially formed nanoceria is often calcined, that is, exposed to temperatures of 400 °C and more. This is appropriate for nanoceria used as a combustion catalyst, as the calcined surface can become quite active when combustion temperatures are reached. Nanoceria with surface hydroxy groups, as expected for solvothermally synthesized nanoceria, are chemically reactive at physiological temperatures. Increased temperature results in water loss as two surface hydroxy groups condense, forming water and a Ce–O–Ce surface site. The amount of surface hydroxy groups decreased as the temperature increased from 200 to 400 °C, with the persistence of some hydroxy groups up to 600 °C [57]. Reports state that NM-212 was prepared by precipitation [7,24], however, the procedures have not been reported to know if it was precipitated from a liquid or gas phase. If NM-212 was exposed to a high temperature it would be expected to have less surface Ce^3+^ [58], which is the case (Supporting Information File 1, Table S1), and show slow dissolution, as was seen with NM-212 and the calcined solvothermally synthesized nanoceria.

The cerium valence state difference on the nanoceria surface may contribute to the dissolution rate difference. As the nanoceria size decreases, the Ce^3+^/Ce^4+^ ratio increases (Figure 13). The edge and core of NM-212 had about 90% Ce^4+^ whereas the 4 nm solvothermally synthesized ceria exhibited predominantly Ce^3+^. Increased Ce^3+^ is accompanied by an increase in oxygen vacancies that weaken the crystal lattice [14]. According to the dissolution framework by Gray and co-workers, Ce^3+^ can follow more dissolution pathways (including oxidative dissolution) than Ce^4+^ [59]. The much greater percentage of Ce^3+^ on the surface of solvothermally synthesized nanoceria may have contributed to its more rapid dissolution. Although the dissolution rates of NM-211 and NM-212 are similar ([60] and Figure 7), NM-211 dissolved faster. There is significantly more Ce^3+^ on the surface of NM-211 than on that of NM-212 [7]. Therefore, among these three nanoceria forms, increased surface Ce^3+^ correlated with a more rapid dissolution.

### Role of phosphate in the fate of cerium/nanoceria

Precipitation and formation of cerium phosphate particles might contribute to the slow clearance of nanoceria from the lung reported in [4,9,51,61]. Cerium ions, introduced as such or released during nanoceria dissolution, in the presence of phosphate form cerium phosphate [48,52,62–64] as was reported after introduction of cerium ions into the lung [65]. After systemic administration of solvothermally synthesized nanoceria, cerium phosphate particles were seen in rat liver and spleen [48,63]. After inhaling 3 mg/m^3^ NM-212 for two years, CeO^+^ and CePO_4_^+^ were seen in rat femur [9]. These studies demonstrate a certain extent of dissolution of both nanoceria forms in vivo. Ce^3+^ phosphate is quite insoluble. Ce^4+^ phosphate is even less soluble (log *K*_sp_ = 10^−26^ to 10^−23^ for Ce(III) phosphate in 0.01 to 12 M H_3_PO_4_, 0.1 M NaH_2_PO_4_, or 0.1 M HCl [66–68]; log *K*_sp_ = 10^−115^ for Ce(IV) phosphate in 0.01 to 12 M H_3_PO_4_ and 10^−34^ at pH 0.4 [66,69]). The present study assessed the hypothesis that phosphate groups not in direct contact with nanoceria would create a sink for released cerium ions and increase the nanoceria dissolution rate. The results do not support the hypothesis. However, while Ce forms a strong complex with orthophosphate anions as indicated by the above *K*_sp_ values, it is possible that the complexation constants for the immobilized phosphate groups on the LN resin and HTP are weaker than for Ce^3+^ and orthophosphate. The results are consistent with a two-step nanoceria dissolution process. First, the cerium ions leave the nanoceria surface, by dissociative dissolution of Ce^4+^ and oxidative or dissociative dissolution of Ce^3+^, consistent with the interpretation that nanoceria dissolution occurs at the solid–liquid interface [14]. Cerium ions may then complex with phosphate. Nanoceria dissolution is pH-dependent ([14,39,70] and Figure 11). Nanoceria dissolution presumably results from an interaction between the hydrogen ion and the nanoceria surface. A hypothesis is that hydrogen “steals” oxygen from the Ce^3+^ of the surface Ce–O complex, enabling Ce^3+^ release. This would be consistent with increased dissolution as the pH value decreases, surface Ce^3+^ increases, and size decreases (and surface Ce^3+^ increases). Also, there is more available oxygen on the surface of nanoceria not exposed to high temperature.

### Biological identity of as-prepared and partially dissolved nanoceria

Nanoceria has the potential to act as a pro- or antioxidant, depending on its valence, and the oxidative stress level of the system under study. Solvothermally synthesized nanoceria increased protein carbonyls in rats and *Caenorhabditis elegans* that were not induced by oxidative stress [71–72]. In models of oxidative stress, nanoceria decreased elevated levels of protein carbonyls in the lung and hippocampus of hypoxic rats [73–74], in the kidney of diabetic mice [75], in murine embryo tissue in a model of gestational diabetes [76], and in the right ventricle of rats with monocrotaline-induced hypertension [77]. It protected against protein carbonyl elevation induced by acrylamide in HepG2 (human liver epithelial) cells [78] and cyclophosphamide in murine testicles [79].

Increased protein carbonyls in RAW 264.7 cells produced by solvothermally synthesized nanoceria are consistent with the ability of nanoceria to act as a pro-oxidant in systems where oxidative stress is at its normal (basal) level. The lower increase of protein carbonyls following the exposure to partially dissolved nanoceria may be due to the increased surface content of Ce^3+^ providing antioxidant potential. After dialysis in water, the partially dissolved nanoceria rapidly precipitates from the dispersion, presumably due to the loss of surface citrate and reduced surface charge. The particles do not pass through a 0.2 micrometer MWCO filter, suggesting that they were quite large, which may have affected cell uptake. The results illustrate an effect of nanoceria dissolution on its biological identity.

### Nanoceria reactivity

Nanoceria reactivity increased as the content of Ce^3+^ on the surface increased and the particle size decreased. The solvothermally synthesized nanoceria exhibited the highest mass-based reactivity, followed by the intermediate sized NM-211 and the largest tested nanoceria with the least amount of surface Ce^3+^, NM-212. As discussed above for the nanoceria dissolution rate, size contributes to the reactivity differences, but is not sufficient to explain them. After normalizing the reactivity to surface-based reactivity, the value for the solvothermally synthesized nanoceria (38 ± 5 nmol TEU/m^2^) was significantly greater than those for the other nanoceria materials (14 ± 1 and 13 ± 1 nmol TEU/m^2^). The difference can be attributed to the chemical identity of the nanoceria surface, where surface Ce^3+^ mediates the redox cycle influencing the reactivity.

## Conclusion

The dissolution study results, when related to the physicochemical properties of the tested nanoceria, indicate that the amount of surface Ce^3+^ is the primary factor influencing solubility. Higher nanoceria preparation temperature reduces the amount of surface Ce^3+^, resulting in reduced solubility. The results suggest that NM-212 was exposed to a high temperature, as is common for nanoceria prepared for industrial applications. The results also show the importance of a complete documentation of the preparation procedures. This is an issue with commercially prepared nanoceria that have been used in many studies, for which the preparation procedures are not available. As it is clear that physicochemical properties of nanomaterials influence their fate and behavior, and the preparation method influences their physicochemical properties, documentation of the preparation, including temperature, is needed to fully interpret the relationship between physicochemical properties of nanoceria and their fate and effects.

## Supporting Information

File 1 Additional experimental data.
