# Supplementary material for: The preparation temperature influences the physicochemical nature and activity of nanoceria
Source: Beilstein J Nanotechnol. 2021 Jun 4;12:525–40. doi: 10.3762/bjnano.12.43 (PMC8182686; doi:10.3762/bjnano.12.43)
Supplement: File 1 — Additional experimental data. [file Beilstein_J_Nanotechnol-12-525-s001.pdf]

BEILSTEIN JOURNAL OF NANOTECHNOLOGY

Supporting Information

for

The preparation temperature influences the physicochemical nature and activity of nanoceria

Robert A. Yokel, Wendel Wohlleben, Johannes Georg Keller, Matthew L. Hancock, Jason M. Unrine, D. Allan Butterfield and Eric A. Grulke

Beilstein J. Nanotechnol. **2021,** *12,* 525–540. doi:10.3762/bjnano.12.43

Additional experimental data

License and Terms: This is a supporting information file under the terms of the Creative Commons Attribution License (https://creativecommons.org/licenses/by/4.0). Please note that the reuse, redistribution and reproduction in particular requires that the author(s) and source are credited and that individual graphics may be subject to special legal provisions.
The license is subject to the *Beilstein Journal of Nanotechnology* terms and conditions: (https://www.beilstein-journals.org/bjnano/terms)

Table S1: Physicochemical properties of NM-212 and the solvothermally synthesized nanoceria.

	NM-212		Solvothermal synthesis-produced	
Metric	Value	Reference	Value	Reference
Mean primary particle Feret diameter by SEM	28 nm	[1]		
Primary particle size, by TEM	<10 to >100 nm	[2]		
Mean primary particle size	40 nm	[3,4]		
Mean primary particle diameter, Feret, and area	22 nm, 29 nm, 484 nm^2	This article	4.2 nm	[5-7]
BET surface area	27 m^2/g	[1-4,8-10]		
Hydrodynamic diameter (and polydispersity index) in water	207 nm (0.196)	[9]	11 nm	[6,7]
Hydrodynamic size (and mean polydispersity) in water	213 nm (0.255)	[2]		
Equivalent spherical diameter in water	135 nm	[2]		
Agglomerate diameter	3000–150,000 nm	[3]		
Agglomerate density	2 g/m^3	[3]		
Dispersion stability (half-life) in water	2676 min	[2]		
Crystallite phase/planes	Crystalline, cerianite like, irregular, roughly globular, ceria-cubic	[2,3,9]	Face-centered cubic fluorite	[6,7]
Shape	Polyhedral	[2]	Hexagonal	[7]
Shape	Mixed spheroidal and platelets	[10]		
Shape	Cubic or triangular	This article		
Purity	>99.5%	[10]		
TGA-induced weight loss	0.7%	[3]	15%	[7]
TGA-induced weight loss	1.3%	This article		
Surface chemistry (3-10 nm depth)	C 79.9%, O 17.7%	[3,4]		
Surface chemistry (0-12 nm depth), by XPS (PROSPEcT)	C 75.2%, O 22.9%, Ce 2%	[2]		

Surface chemistry (0-10 nm depth) XPS	C 31.9%,<br>O 42.1%,<br>Ce 25.7%	[2]		
Surface chemistry pelleted, XPS (JRC)	C 19.5%,<br>O 66.9%,<br>Ce 13.6%	[2]		
Surface chemistry, FTIR	Peaks at 1630, 1420, and 1320 cm^{-1}	This article		
Elemental mapping (EDS)	Majority Ce and O, with small % C and Na	This article		
Particle coating			Citrate (monolayer)	[7]
Zeta potential in water	42 mV at pH 7, positive over entire physiological range	[3,9]		
Zeta potential in water	33 mV	[2]		
Zeta potential	15 mV at pH 7	[4]	−40 mV at pH 7	[7]
Zeta potential	42 mV at pH 7	[3]		
IEP	above pH 10 (always cationic)	[3]	pH 1.41	[7]
Hydrophobicity (water contact angle)	60	[4]		
Oxidation state (within the XPS-accessible surface layer)	Ce^{3+} 14%	[3,9]	Core primarily Ce^{4+}, surface predominantly Ce^{3+}	[5-7]
Oxidation state	Ce^{3+} 6.9%, Ce^{4+} 93.1%	[2]		
Oxidation state (particle edge)	ca. 90% Ce^{4+}	This article		
Oxidation state (particle center)	ca. 90% Ce^{4+}	This article		
Surface reactivity, as photon efficiency	1.3×10^{-2}	[3,9]		
TEM image of dry powder	Figure 1B	[1]	Figure 1-1<br>Figure 1	[7]<br>[6]
SEM image of dry powder	Figure 1E	[1]		
HRTEM/STEM image of primary crystallite, primary particles, and their agglomerate			Figure 1	[5]
TEM image of primary particles	Figure 1	[9]		
TEM image of primary particles	Figures 17 & 18 show irregular,	[2]		

	non-homogeneous particles			
TEM image of primary particles	Figure 1	This article		
SEM image of dry powder	Figure 2b	[3]		
SEM image of dry powder	Figure 1b	[9]		
SEM image of dry powder	Figure 2	[2]		
TEM image before and after 7-day incubation in phagolysosomal simulant fluid	Figure 4d	[10]		
TEM image after 28-day incubation in phagolysosomal simulant fluid	Figure 3b	[3]		
SEM image after 28-day incubation in phagolysosomal simulant fluid	Figure 2c	[9]		
Selected area diffraction after 28-day incubation in phagolysosomal simulant fluid	Figure 3b	[3]		

Table S2: Reports of nanoceria solubility.

Dissolution	Solubilizing media/conditions	Physicochemical identity (average primary particle size in nm), ((average hydrodynamic size in nm))	Reference
No significant dissolution	72 h at 37 °C in pH 7.0 physiological saline	(7), ((13)), specific surface area 87 m^2/g	[11]
No significant dissolution	72 h at 37 °C in pH 4.5 artificial phagolysosomal fluid		
1.3%, 1.8%, 0.8%, and 0.9%	24 h in pH 7.8 DMEM + 10% FBS	(7), BET 63 m^2/g; (7), BET 44 m^2/g; (7), BET 38 m^2/g; and (7), BET 63 m^2/g	[12]
3% and 0.08%	28 d in pH 4 or 7 artificial soil solution	(8 to 9)	[13]
2.3%, 0.3%, and 0.06%	28 d in pH 4, 7, or 9, 0.1 M NaCl	(8 to 9), ((130 at pH 4.5; >1000 at pH 7, and 300 at pH 9))	
No significant dissolution	24 h at room temperature in pH 7.4 artificial interstitial fluid	(4), ((132)), surface area 66 m^2/g; and (10), ((88)), surface area 24 m^2/g	[14]
0.2% and 0.3%	24 h at room temperature in pH 5.5 artificial lysosomal fluid		
Extremely poor	Water at neutral pH	(45) (NM-211); (28), **(NM-212);** and (615), (NM-213)	[1]
No dissolution	24 h in DMEM-FBS	(14), specific surface area 84 m^2/g	[15]
<0.001%	24 h at 25 °C in water	(10 to 200), ((>10,000)), specific surface area 33 m^2/g	[16]
<0.001%	24 h at 25 °C in DMEM + 10% fetal calf serum		
Extremely small solubility	At 37 °C in DMEM + 10% FBS	(8), ((669 in PBS and 191 in DMEM + 10% FBS)); (20), ((35 in PBS and 61 in DMEM + 10% FBS))	[17]
Insignificant	Exposure to artificial daylight, initially at pH 8.5 and at pH 6.2 after 112 d	(4)	[18]
<0.001%, 0.002%	28 d in water	NM-211 and **NM-212**	[3,9]
<0.001%, <0.001%	28 d in DMEM + fetal calf serum		
<0.001%, <0.001%	28 d in phagolysosomal simulant fluid		
<0.001%	28 d in PBS		[3,9]

<0.001%	7 and 28 d in fasted state simulated intestinal fluid	**NM-212**	
0.02%	1 and 28 d in 0.1 N HCl		
5%, 8%, and 18% in 1- and 50 mM ascorbate and 10 mM glutathione	CeO_2-coated mesoporous silica 3 d with 1, 5, 10, or 50 mM ascorbate or 1, 5, or 10 mM glutathione	(4)	[19]
Greater for smaller ENMs, at pH 4 than 6, and in presence of gum arabic and absence of KH_2PO_4	7 d in 20% Hoagland solution at pH 4 or 6, and added gum arabic or phosphate deletion	(8.9), ((140)), BET 233 g/m^2; (22.8), ((129)), BET 22.4 g/m^2; (63.9) ((1182)) BET 4.9 g/m^2	[20]
No dissolution	21 d in DI water, fish medium (pH 7.3), daphnia medium (pH 7.9), or seawater (pH 8.8)	NM-211, **NM-212**, and NM-213	[2]
<0.0025% for NM-211, NM-212, 0.3% for NM-213	72 h at 24 °C in pH 6.5 synthetic soft water		
Solubility constant (K_{sp})	0.13		[21]
Dissolution rate: 0.0576 and 0.0741 mmol·kg^{-1}·h^{-1}	120 h in pH 1.65 in 0.01 mM $NaNO_3$	Uncapped (32) and (78), BET surface area 40 and 11.5 m^2/g	[22]
Dissolution rate: 0.0362 and 0.0209 mmol·kg^{-1}·h^{-1}	120 h in pH 1.65 in 0.01 mM $NaNO_3$ and 0.1 mM phosphate		
Dissolution rate: 0.0168 and 0.0177 mmol·kg^{-1}·h^{-1}	120 h in pH 4.5 in 0.01 mM $NaNO_3$ and 0.2 M Na acetate		
Dissolution rate: 0.0057 and 0.0084 mmol·kg^{-1}·h^{-1}	120 h in pH 4.5 in 0.01 mM $NaNO_3$, 0.2 M Na acetate, and 0.1 mM phosphate		
No detectable dissolution	120 h in pH 7.5		
No detectable dissolution	120 h in pH 12.4		
0.4% in water, + PO_4 0%, 14% in citric and ascorbic acids, + PO_4 0.3%, 3.7% in citric acid and catechol, + PO_4 1.9%	21 d in pH 5.5 with 1 mM citric and ascorbic acid or citric acid and catechol, and KH_2PO_4	(25), ((123 in water, 1235 in organic solution, and 1485 in organic solution and phosphate))	[23]
0% lung, ~0.2% intestinal, ~0.2% gastric IVC method, 5.5% gastric SBRC method	24 h at 37 °C in simulated lung (pH 4.5 and 7.3), 4 h intestinal, and 2 h gastric (pH 1.8 IVG and 1.5 SBRC) fluids		[24]

<3%	7 d at 37 °C in pH 4.55 phagolysosomal simulant fluid	NM-211, **NM-212**	[10]
Extremely low solubility	30 m in MES buffer at pH 4.5, 5,5. 6.5, or 7.5	(18), ((542 in water and 250 in DMEM)), zeta 22 mV in water and -15 mV in DMEM	[25]
No detectable dissolution	96 h in water	(24), ((~ 3000 in water))	[26]
1.1% and 0.19%	7 d at 37 °C in pH 4.55 phagolysosomal simulant fluid	NM-211, **NM-212**	[27]
0.06 $ng/cm^2/h$	Lysosomal dissolution rate		[4]
0.3% in medium, no increase with 0.01 M HCl or 1 mM citric acid, 0.9% in ascorbic acid, 44% in ascorbic and citric acids	3 h in pH 7.1 Luria-Bertani medium, with 0.01 M HCl (not pH 7.1), 1 mM ascorbic and/or 1 mM citric acid	(8 x 168 rods)	[28]
Dissolution half-life 58,200 h	28 weeks in pH ~ 6 iso-osmotic water	**Solvothermal synthesis-produced nanoceria of this report**	[5,29]
	28 weeks in pH ~ 4.5 iso-osmotic water		
Carboxylic acid-dependent dissolution half-life 800 to 3150 h	28 to 30 weeks at 37 °C in pH 4.5 iso-osmotic 110 mM acetic, adipic, citric, glutaric, DL-3-hydroxybutyric, lactic, DL-malic, pimelic, succinic acid, or tricarballylic acid		
Dissolution half-life 7490 h	28 weeks in 20 mM pH 4.5 iso-osmotic ammonium		

References

1. Geraets, L.; Oomen, A. G.; Schroeter, J. D.; Coleman, V. A.; Cassee, F. R. *Toxicol. Sci.* **2012,** *127,* 463–473. doi:10.1093/toxsci/kfs113
2. Singh, C.; Friedrichs, S.; Ceccone, G.; Gibson, N.; Jensen, K. A.; Levin, M.; Infante, H. G.; Carlander, D.; Rasmussen, K. *Cerium Dioxide, NM-211, NM-212, NM-213. Characterisation and test item preparation;* Publications Office of the European Union, 2014. doi:10.2788/80203
3. Keller, J.; Wohlleben, W.; Ma-Hock, L.; Strauss, V.; Gröters, S.; Küttler, K.; Wiench, K.; Herden, C.; Oberdörster, G.; van Ravenzwaay, B.; Landsiedel, R. *Arch. Toxicol.* **2014,** *88,* 2033–2059. doi:10.1007/s00204-014-1349-9
4. Wohlleben, W.; Hellack, B.; Nickel, C.; Herrchen, M.; Hund-Rinke, K.; Kettler, K.; Riebeling, C.; Haase, A.; Funk, B.; Kühnel, D.; Göhler, D.; Stintz, M.; Schumacher, C.; Wiemann, M.; Keller, J.; Landsiedel, R.; Brossell, D.; Pitzko, S.; Kuhlbusch, T. A. J. *Nanoscale* **2019,** *11,* 17637–17654. doi:10.1039/C9NR03306H
5. Yokel, R. A.; Hancock, M. L.; Grulke, E. A.; Unrine, J. M.; Dozier, A. K.; Graham, U. M. *Nanotoxicology* **2019,** *13,* 455–475. doi:10.1080/17435390.2018.1553251
6. Yokel, R. A.; Tseng, M. T.; Butterfield, D. A.; Hancock, M. L.; Grulke, E. A.; Unrine, J. M.; Stromberg, A. J.; Dozier, A. K.; Graham, U. M. *Nanotoxicology* **2020,** *14,* 827–846. doi:10.1080/17435390.2020.1770887
7. Hancock, M. L.; Yokel, R. A.; Beck, M. J.; Calahan, J. L.; Jarrells, T. W.; Munson, E. J.; Olaniyan, G. A.; Grulke, E. A. *Appl. Surf. Sci.* **2021,** *535,* 147681. doi:10.1016/j.apsusc.2020.147681
8. Gosens, I.; Mathijssen, L. E. A. M.; Bokkers, B. G. H.; Muijser, H.; Cassee, F. R. *Nanotoxicology* **2014,** *8,* 643–653. doi:10.3109/17435390.2013.815814
9. Molina, R. M.; Konduru, N. V.; Jimenez, R. J.; Pyrgiotakis, G.; Demokritou, P.; Wohlleben, W.; Brain, J. D. *Environ. Sci.: Nano* **2014,** *1,* 561–573. doi:10.1039/C4EN00034J

10. Koltermann-Jülly, J.; Keller, J. G.; Vennemann, A.; Werle, K.; Müller, P.; Ma-Hock, L.; Landsiedel, R.; Wiemann, M.; Wohlleben, W. *NanoImpact* **2018,** *12,* 29–41. doi:10.1016/j.impact.2018.08.005
11. He, X.; Zhang, H.; Ma, Y.; Bai, W.; Zhang, Z.; Lu, K.; Ding, Y.; Zhao, Y.; Chai, Z. *Nanotechnology* **2010,** *21,* 285103/1–285103/8. doi:10.1088/0957-4484/21/28/285103
12. Kroll, A.; Dierker, C.; Rommel, C.; Hahn, D.; Wohlleben, W.; Schulze-Isfort, C.; Gobbert, C.; Voetz, M.; Hardinghaus, F.; Schnekenburger, J. *Part. Fibre Toxicol.* **2011,** *8,* 9. doi:10.1186/1743-8977-8-9
13. Cornelis, G.; Ryan, B.; McLaughlin, M. J.; Kirby, J. K.; Beak, D.; Chittleborough, D. *Environ. Sci. Technol.* **2011,** *45,* 2777–2782. doi:10.1021/es103769k
14. Cho, W.-S.; Duffin, R.; Thielbeer, F.; Bradley, M.; Megson, I. L.; MacNee, W.; Poland, C. A.; Tran, C. L.; Donaldson, K. *Toxicol. Sci.* **2012,** *126,* 469–477. doi:10.1093/toxsci/kfs006
15. Horie, M.; Fujita, K.; Kato, H.; Endoh, S.; Nishio, K.; Komaba, L. K.; Nakamura, A.; Miyauchi, A.; Kinugasa, S.; Hagihara, Y.; Niki, E.; Yoshida, Y.; Iwahashi, H. *Metallomics* **2012,** *4,* 350–360. doi:10.1039/c2mt20016c
16. Wohlleben, W.; Ma-Hock, L.; Boyko, V.; Cox, G.; Egenolf, H.; Freiberger, H.; Hinrichsen, B.; Hirth, S.; Landsiedel, R. *J. Ceram. Sci. Technol.* **2013,** *4,* 93–104. doi:10.4416/JCST2012-00045
17. Zhou, X.; Wang, B.; Chen, Y.; Mao, Z.; Gao, C. *J. Nanosci. Nanotechnol.* **2013,** *13,* 204–215. doi:10.1166/jnn.2013.6788
18. Auffan, M.; Masion, A.; Labille, J.; Diot, M.-A.; Liu, W.; Olivi, L.; Proux, O.; Ziarelli, F.; Chaurand, P.; Geantet, C.; Bottero, J.-Y.; Rose, J. *Environ. Pollut. (Oxford, U. K.)* **2014,** *188,* 1–7. doi:10.1016/j.envpol.2014.01.016
19. Muhammad, F.; Wang, A.; Qi, W.; Zhang, S.; Zhu, G. *ACS Appl. Mater. Interfaces* **2014,** *6,* 19424–19433. doi:10.1021/am5055367
20. Schwabe, F.; Schulin, R.; Rupper, P.; Rotzetter, A.; Stark, W.; Nowack, B. *J. Nanopart. Res.* **2014,** *16,* 2668. doi:10.1007/s11051-014-2668-8
21. Dahle, J. T.; Arai, Y. *Int. J. Environ. Res. Public Health* **2015,** *12,* 1253–1278. doi:10.3390/ijerph120201253

22. Dahle, J. T.; Livi, K.; Arai, Y. *Chemosphere* **2015,** *119,* 1365–1371. doi:10.1016/j.chemosphere.2014.02.027
23. Rui, Y.; Zhang, P.; Zhang, Y.; Ma, Y.; He, X.; Gui, X.; Li, Y.; Zhang, J.; Zheng, L.; Chu, S.; Guo, Z.; Chai, Z.; Zhao, Y.; Zhang, Z. *Environ. Pollut.* **2015,** *198,* 8–14. doi:10.1016/j.envpol.2014.12.017
24. Zhong, L.; Yu, Y.; Lian, H.-z.; Hu, X.; Fu, H.; Chen, Y.-j. *J. Nanopart. Res.* **2017,** *19,* 1–10. doi:10.1007/s11051-017-4064-7
25. Mirshafiee, V.; Sun, B.; Chang, C. H.; Liao, Y.-P.; Jiang, W.; Jiang, J.; Liu, X.; Wang, X.; Xia, T.; Nel, A. E. *ACS Nano* **2018,** *12,* 3836–3852. doi:10.1021/acsnano.8b01086
26. Zhang, Y.; Blewett, T. A.; Val, A. L.; Goss, G. G. *Environ. Sci.: Nano* **2018,** *5,* 476–486. doi:10.1039/C7EN00842B
27. Koltermann-Jülly, J.; Keller, J. G.; Vennemann, A.; Werle, K.; Müller, P.; Ma-Hock, L.; Landsiedel, R.; Wiemann, M.; Wohlleben, W. *NanoImpact* **2019,** *14,* 100154. doi:10.1016/j.impact.2019.100154
28. Xie, C.; Zhang, J.; Ma, Y.; Ding, Y.; Zhang, P.; Zheng, L.; Chai, Z.; Zhao, Y.; Zhang, Z.; He, X. *Environ. Sci.: Nano* **2019,** *6,* 216–223. doi:10.1039/C8EN01002A
29. Grulke, E. A.; Beck, M. J.; Yokel, R. A.; Unrine, J. M.; Graham, U. M.; Hancock, M. L. *Environ. Sci.: Nano* **2019,** *6,* 1478–1492. doi:10.1039/C9EN00222G
